# A GUV-based assay to reconstitute membrane tethering in vitro

**DOI:** 10.1091/mbc.E25-11-0531

**Published:** 2026-06-10

**Authors:** Devika Andhare, Michael J. Ragusa

**Affiliations:** ^a^Department of Chemistry, Dartmouth College, Hanover, NH 03755; University of California, Berkeley

## Abstract

Membrane tethering is essential for the generation of organelle contact sites, the catabolic process of autophagy, and to anchor incoming vesicles to their target membranes before vesicle fusion. Although membrane tethering is critical for cellular function, many of the current biochemical techniques to test for membrane tethering rely on indirect readouts and are limited in their ability to monitor protein localization at sites of tethering. As such, we recently developed a fluorescence microscopy-based giant unilamellar vesicle and liposome tethering assay (GLT) to study the membrane tethering properties of two autophagy proteins. In this study, we used GLT with engineered membrane tethers to demonstrate the ease of use, methods of analysis, versatility, and sensitivity of the assay. We demonstrate that: 1) GLT can be used to study liposome tethering, fusion, and phosphatase-mediated detethering of tethered liposomes, 2) GLT detects tethering with comparable sensitivity with less direct methods for monitoring membrane tethering while allowing simultaneous monitoring of membrane and protein localization, and 3) GLT can be used to monitor the kinetics of membrane tethering in real time. Collectively, our results demonstrate GLT is a broadly useful method to study membrane tethering in vitro.

## Abstract (executive summary)

- Membrane tethering is critical for organelle contacts and vesicle trafficking. However, studying membrane tethering in vitro often relies on indirect measures of membrane tethering.
- The authors used a new assay that combines giant unilamellar vesicles and liposomes to monitor membrane tethering in vitro. They demonstrate that this assay allows real-time, simultaneous monitoring of membrane tethering, protein localization, and fusion of tethered membranes.
- These results suggest that this new assay will be broadly useful to study a variety of membrane tethering events, including both homotypic and heterotypic tethering events, and will complement existing methods.

## INTRODUCTION

Over the last two decades, an increasing number of studies have highlighted the importance of membrane tethering events in cells (Szentgyörgyi and Spang, 2023). Membrane tethering occurs when individual proteins or multiprotein complexes bridge two different membranes, holding them within a defined distance, frequently less than 100 nm. These tethering events can be homotypic, where both membrane compositions are identical, or more commonly heterotypic, where two distinct membranes, such as the plasma membrane (PM) and the endoplasmic reticulum (ER), are tethered (Scorrano *et al.*, 2019). The main functions of membrane tethering in cells are to generate organelle contact sites and to recruit vesicles to their target organelles before fusion (Phillips and Voeltz, 2016; Ungermann and Kümmel, 2019).

Due to the diversity of membrane tethering events in cells, the proteins required for tethering vary dramatically in their size, domain architecture and mechanism by which they tether membranes. One of the most direct membrane tethers is the extended synaptotagmin (E-synt) family of proteins that functions to tether the PM to the ER by directly interacting with both membranes (Saheki and De Camilli, 2017; Bian *et al.*, 2018). However, most membrane tethering events require at least two proteins to bridge the membranes, and many tethering events require larger multiprotein complexes (Scorrano *et al.*, 2019; Szentgyörgyi and Spang, 2023). For example, in vesicle fusion, large multisubunit tethering complexes, like the HOPS and CORVET complexes, bind to RAB GTPases, which are anchored to the membranes by posttranslational modifications, and SNARE proteins, embedded in membranes, to facilitate tethering (Jahn and Scheller, 2006; Spang, 2016; Lurick *et al.*, 2018; Ungermann and Kümmel, 2019).

Given the diversity of tethering events and proteins that facilitate these events, studying membrane tethering has been challenging. This is particularly true in cells due to the complexity of contact sites, the transient nature of tethering in vesicular trafficking, and the indirect nature of the available assays (Huang *et al.*, 2020). Fluorescently labeled proteins and bimolecular fluorescence complementation have been used to determine if specific proteins or protein complexes are located at potential tethering sites in cells (Huang *et al.*, 2020; Jing *et al.*, 2020). However, it is unclear whether these proteins are actively participating in tethering or whether they are localized to these sites for other purposes. Disrupting potential tethers in cells may not lead to a loss in the contact site due to redundancy in tethers at the site (Eisenberg-Bord *et al.*, 2016). In addition, a loss of contact sites or fusion events in response to protein depletion or knockout could be due to indirect effects and not necessarily due to direct loss of membrane tethering. Instead, biochemical approaches have been critical to determine whether a protein or a protein complex is capable of mediating membrane tethering. For example, biochemical studies have demonstrated the importance of RABs and SNAREs for HOPS-mediated homotypic tethering events (Stroupe *et al.*, 2009; Shvarev *et al.*, 2022). Although these assays have been essential to shed light on the mechanisms of membrane tethering, many commonly used biochemical assays rely on indirect measures of tethering. This includes light scattering, turbidity assays, and microscopy of liposome clustering, which all monitor the increasing size of vesicle clusters as they are tethered rather than two differentially labeled membranes being held together, and can yield false positives due to nonspecific aggregation of liposomes and/or protein (Ueda *et al.*, 2020).

To investigate membrane tethering in vitro, we wanted to create an assay capable of reconstituting and examining the diverse spectrum of membrane tethering events that occur in cells. Additionally, we sought to establish an assay that would be easy to perform and analyze, provide a more direct readout than existing assays, and enable independent monitoring of two distinct membranes while simultaneously tracking protein recruitment to these membranes. This led to the development of the Giant unilamellar vesicle (GUV) and liposome tethering (GLT) assay that we recently used in two separate studies to evaluate the membrane tethering capabilities of the autophagy proteins Atg23 and Atg11 (Hawkins *et al.*, 2022; Andhare *et al.*, 2026). Here, we focus exclusively on the GLT assay and its applications. We demonstrate that GLT can be performed with standard lab equipment, yields data that are straightforward to analyze, and achieves comparable sensitivity to established tethering assays. Importantly, GLT provides unique advantages over existing membrane tethering assays, including the ability to simultaneously monitor protein localization and tethering, as well as to distinguish membrane binding from tethering in a single experiment. Additionally, GLT enables real-time analysis of tethering kinetics in vitro and can be adapted to study membrane detethering and fusion, further adding to its versatility. Based on these results, we anticipate that GLT will find broad applicability in the biochemical investigation of membrane tethering events.

## RESULTS

### Fluorescence-microscopy-based GLT assay

**FIGURE 1:**
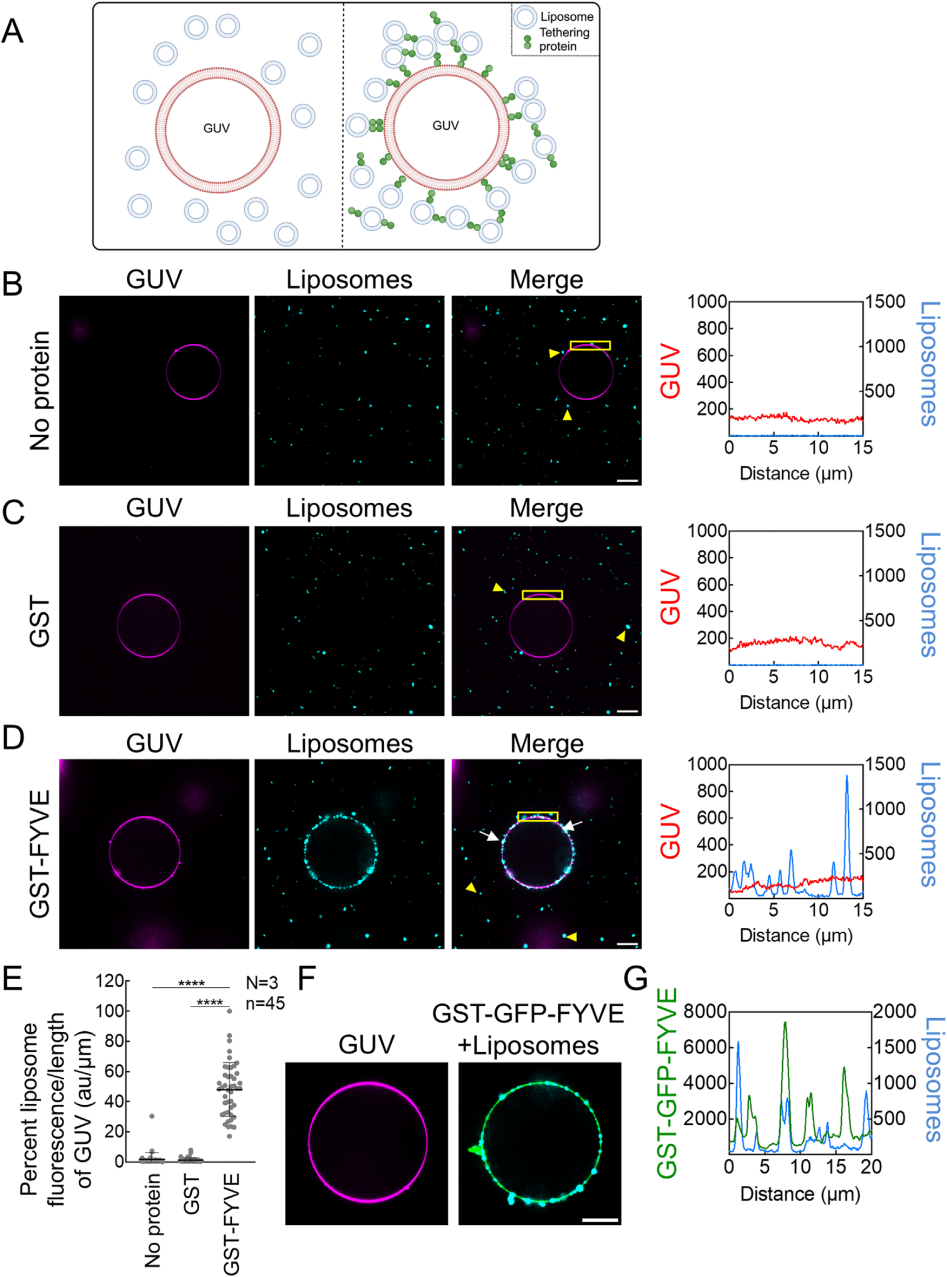
GLT using an engineered tether. (A) Schematic showing the fluorescence microscopy-based GLT assay. GUVs and liposomes containing different fluorophores are mixed with candidate tethering proteins or protein complexes capable of binding and bridging both membranes. Tethering can be directly monitored by analyzing the localization of liposomes along the surface of the GUV. This figure was made in BioRender. Representative images showing 400-nm–extruded liposomes mixed with GUVs (B), GUVs and GST (C), or GUVs and GST-FYVE (D). Merge images show yellow arrowheads, indicating free liposomes and white arrows, indicating tethered liposomes. Fluorescence intensity profiles (from the area highlighted in the yellow box) for the GUV and liposome channels along the circumference of the GUV are shown to the right of each representative image. (E) Scatter plots showing the percentage of liposome fluorescence per unit length of GUV normalized to the largest signal from A to C. Data from three independent repeats (*N* = 3) were pooled and analyzed using nonparametric Mann–Whitney's test. ****p < 0.0001. *n* indicates the total number of GUVs analyzed for each condition. (F) Representative fluorescence images showing GUVs containing PI3P mixed with GST-GFP-FYVE, followed by incubation with PI3P-containing liposomes. (G) Fluorescence intensity profiles for the liposome and protein fluorescence channels around the circumference of the GUV are shown in F. GUVs were made with 74.9 mol% DOPC, 20 mol% DOPS, 5 mol% PI3P, and 0.1 mol% rhodamine PE (RhPE). Liposomes were made with 74.9 mol% DOPC, 20 mol% DOPS, 5 mol% PI3P, and 0.1 mol% of the fluorescent lipophilic dye DiD; scale bar, 10 µm.

To establish an assay to study membrane tethering in vitro, we added extruded liposomes to GUVs labeled with different fluorophores in the presence of a potential tethering protein (Figure 1A). In the absence of a tethering protein or if the protein is incapable of tethering, we anticipate that liposomes will be randomly distributed in the field of view. However, in the presence of a protein capable of tethering membranes, we anticipate that liposomes will be preferentially localized to the circumference of GUVs. We reasoned that the large size of GUVs (typically, 1–100 µm in diameter) would allow us to directly visualize liposomes tethered on the GUV surface, thus eliminating ambiguities arising due to aggregation of liposomes and/or protein. Moreover, the large size of GUVs would also allow us to observe whether a fluorescently tagged protein localizes at tethering sites.

To demonstrate the feasibility of this assay for studying membrane tethering in vitro, we engineered an artificial membrane tether by fusing the monomeric phosphatidylinositol-3-phosphate(PI3P)-binding EEA1 FYVE_1337-1411_ with GST (GST-FYVE) (Dumas *et al.*, 2001). Because GST dimerizes, this generates a stable dimer of EEA1 FYVE_1337-1411_ with two PI3P-binding sites in a single protein complex. Similarly engineered tethers generated by linking membrane-binding domains to GST have been shown to facilitate tethering (Song and Wickner, 2019). To perform GLT, GUVs containing PI3P were first incubated with GST-FYVE, followed by incubation with 400-nm liposomes containing an identical lipid composition to the GUVs except for a different fluorophore for detection. GUVs mixed with PI3P-liposomes alone or with the addition of purified GST showed virtually no colocalization between GUVs and liposomes, demonstrating that the GLT assay has a very low background signal (Figure 1, B and C). In contrast, addition of PI3P-liposomes to GUVs preincubated with 1 µM GST-FYVE showed robust recruitment of liposomes to GUVs as illustrated by fluorescence intensity traces along the surface of a GUV in the liposome fluorescence channel (Figure 1D). This demonstrates that GST-FYVE–mediated homotypic membrane tethering occurred as expected and led to a strong and clear readout in the GLT assay.

To quantify membrane tethering in the GLT assay, we measure liposome fluorescence along the circumference of individual GUVs (Supplemental Figure S1). Images are processed in ImageJ, where background subtraction is performed for the liposome channel using the mode pixel intensity of the whole image. For each GUV, the circumference is outlined with a segmented line (3 pixels wide), and liposome fluorescence intensity is measured. Liposome intensity can be normalized either to the GUV circumference, yielding liposome fluorescence per micron, or to the fluorescence of the tethering protein if it is fluorescently labeled, providing a ratio of liposome to protein fluorescence. At least 30 to 40 GUVs are analyzed across three independent experiments, and the resulting values are plotted to display the full distribution.

Comparison of liposome fluorescence per micron calculated along the surface of GUVs further shows that tethering of liposomes to GUVs only occurred in the presence of GST-FYVE thereby validating GLT for the study of membrane tethering (Figure 1E). This is consistent with our previous results that demonstrated that the autophagy protein Atg23 could mediate homotypic tethering and highlights the usefulness of GLT for monitoring homotypic tethering (Hawkins *et al.*, 2022).

To determine whether protein localization could be monitored simultaneously with membrane tethering, we inserted a GFP tag between the GST and FYVE domains in our construct, generating GST-GFP-FYVE. Repeating the assay with GST-GFP-FYVE, we observed that the protein coats GUVs and tethers liposomes (Figure 1F). Fluorescence intensity traces along the circumference of GUVs revealed that some areas with increased liposome fluorescence also showed increased fluorescence of GST-GFP-FYVE, demonstrating that the protein is localized at these sites (Figure 1G). Despite this similarity, some areas of the GFP and liposome fluorescence intensity profiles do not contain overlapping peaks. This is likely due to the ability of GST-GFP-FYVE to bind both GUVs and liposomes that could result in many copies of GST-GFP-FYVE binding to liposomes or GUVs without interacting with the other membrane. It is possible that more specific membrane tethers, which require multiple proteins to bridge membranes with distinct lipid composition, may show a higher correlation between protein and tethering sites. Nevertheless, these results demonstrate that protein localization can be monitored simultaneously with membrane tethering, which is not possible with other established techniques.

### Tethering in the GLT assay is lipid-specific and reversible

**FIGURE 2:**
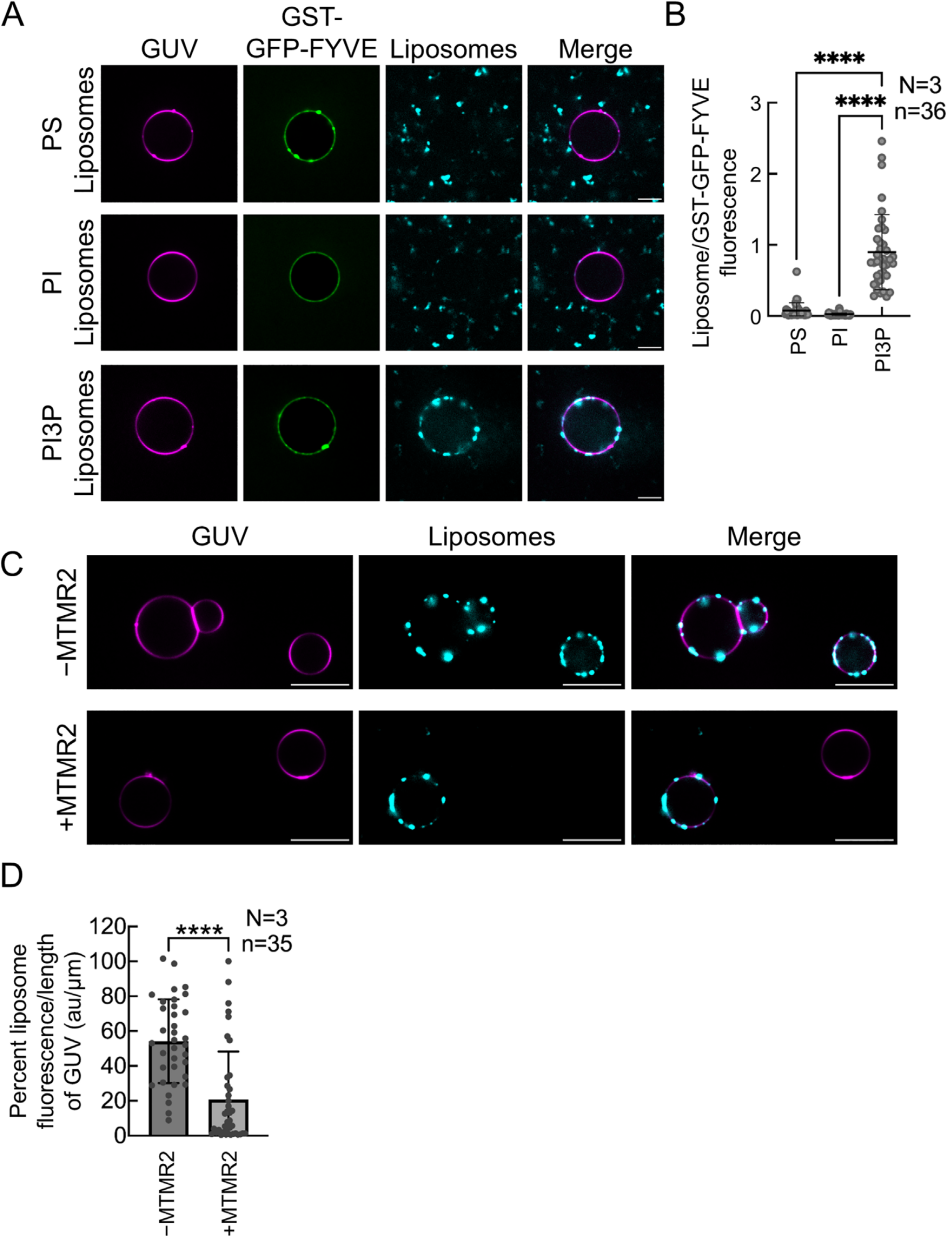
GLT exhibits lipid-specific and reversible tethering. GUVs containing PI3P were incubated with 0.5 µM GST-GFP-GYVE and subsequently mixed with liposomes containing PS, PI, or PI3P. (A) Representative images of GUVs mixed with liposomes containing PS (top), PI (middle), or PI3P (bottom). Merge images show the overlay of GUV and liposome channels; scale bar, 5 µm. (B) Quantification of panel A. Data from three independent experiments (*N* = 3) were pooled and analyzed using one-way ANOVA with the Kruskal–Wallis test. ****p < 0.0001. GUV lipid composition: 74.9 mol% DOPC, 20 mol% DOPS, 5 mol% PI3P, and 0.1 mol% RhPE. PS liposome composition: 69.9 mol% DOPC, 30 mol% DOPS, and 0.1 mol% DiD. PI liposome composition: 69.9 mol% DOPC, 20 mol% DOPS, 10 mol% DOPI, and 0.1 mol% DiD. PI3P liposome composition: 74.9 mol% DOPC, 20 mol% DOPS, 5 mol% PI3P, and 0.1 mol% DiD. (C) Representative fluorescence images showing distribution of liposomes on GUVs in the presence of GST-FYVE before (top panel) and after (bottom panel) incubation with the phosphoinositide phosphatase MTMR2. (D) Quantification of panel C. Data from three independent experiments (*N* = 3) were pooled and analyzed using nonparametric Mann–Whitney's test. ****p < 0.0001. GUVs were made with 74.9 mol% DOPC, 20 mol% DOPS, 5 mol% PI3P, and 0.1 mol% rhodamine PE (RhPE). Liposomes were made with 74.9 mol% DOPC, 20 mol% DOPS, 5 mol% PI3P, and 0.1 mol% of the fluorescent lipophilic dye DiD; scale bar, 10 µm.

To determine whether membrane tethering by GST-FYVE is due to a specific interaction of the FYVE domain with the headgroup of PI3P, we repeated GLT using liposomes containing three different phospholipid compositions. The liposomes contained 69.9% PC and 0.1% RhPE with either 30% PS (PS liposomes), 20% PS and 10% PI (PI liposomes), or 20% PS, 5% PI3P and an additional 5% PC (PI3P liposomes) (Figure 2A). The FYVE domain exhibited significantly higher tethering of PI3P-containing liposomes compared with the other two liposome types, indicating that the tethering by GST-FYVE in the GLT assay is lipid-specific (Figure 2B). Moreover, membrane tethering by GST-FYVE was reversible, as addition of the phosphoinositide phosphatase MTMR2 led to the loss of tethered liposomes from a subset of GUVs (Figure 2C) (Begley *et al.*, 2003; Schaletzky *et al.*, 2003). Consistently, liposome fluorescence per unit length of GUV had a greater than 2.5-fold decrease following the addition of MTMR2. Furthermore, many GUVs had essentially no liposome fluorescence that was not observed before the addition of MTMR2 (Figure 2D). Taken together, this suggests that the GLT assay can be used to monitor both tethering and detethering of liposomes.

### The sensitivity of GLT is comparable with commonly used tethering assays

**FIGURE 3:**
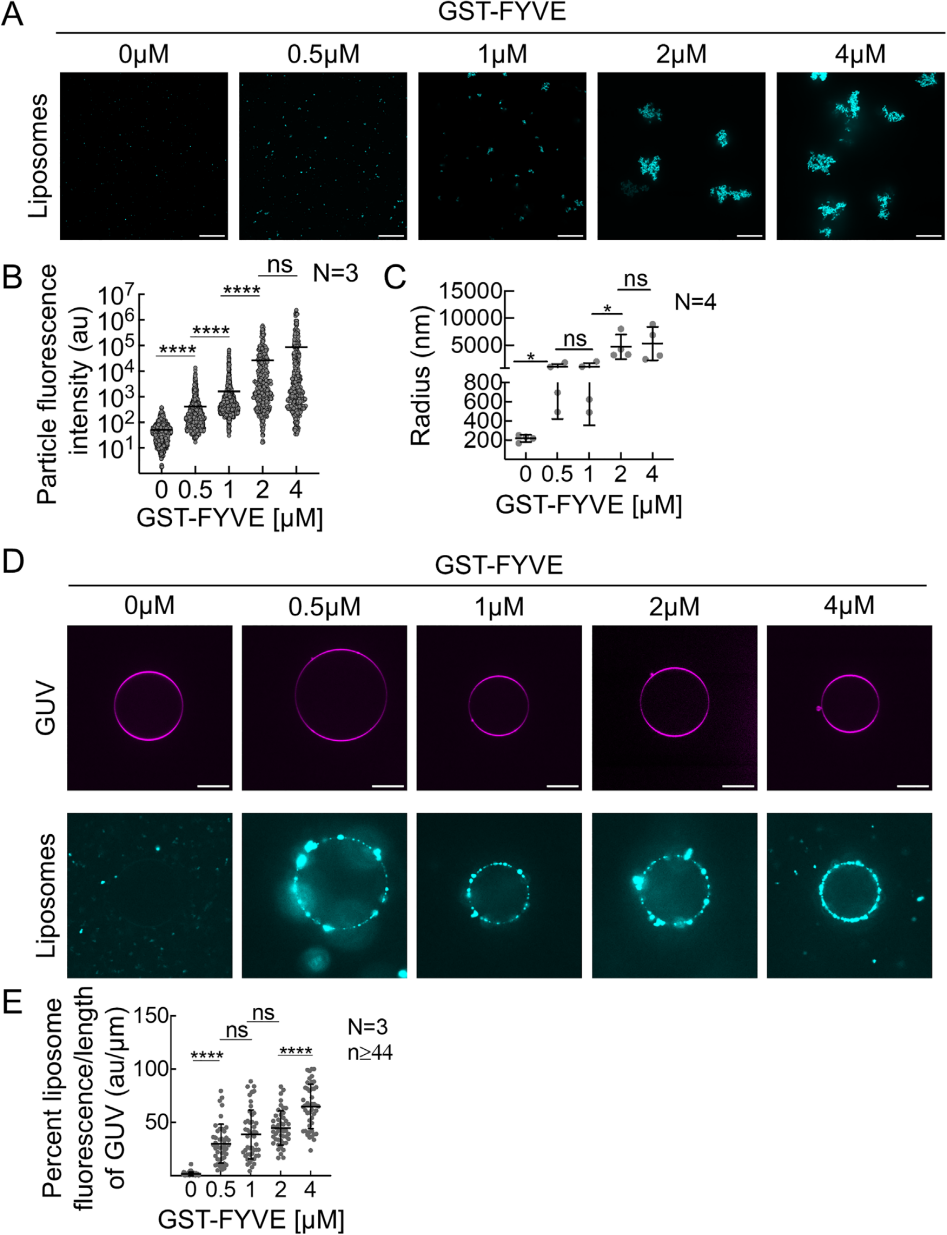
Comparison of GLT with two commonly used methods to study tethering. 400-nm–extruded liposomes containing 74.9 mol% DOPC, 20 mol% DOPS, 5 mol% PI3P, and 0.1 mol% of the fluorescent lipophilic dye DiD were incubated with increasing concentrations of GST-FYVE. Tethering was measured using fluorescence microscopy (A and B) or DLS (C). (A) Representative fluorescence images showing clustering of PI3P-liposomes with increasing concentration of GST-FYVE; scale bar, 10 µm. (B) Quantitation of A. Data from three independent repeats (*N* = 3) were pooled and analyzed using Mann–Whitney's test. ****p < 0.0001. (C) Quantitation of the increase in PI3P-liposome size in response to increasing GST-FYVE concentration as measured by DLS. Data from four independent repeats (*N* = 4) were analyzed using Mann–Whitney's test. *p = 0.0286. (D) Representative fluorescence images from GLT using liposomes as in A and GUVs containing 74.9 mol% DOPC, 20 mol% DOPS, 5 mol% PI3P, and 0.1 mol% RhPE in response to increasing concentration of GST-FYVE. (E) Quantitation of D. Data from three independent repeats (*N* = 3) were pooled and analyzed using Mann–Whitney's test. ****p < 0.0001. *n* indicates the total number of GUVs analyzed per condition.

Having demonstrated that GLT can be used to monitor homotypic membrane tethering using model tethers, we next sought to compare the sensitivity of this assay with some commonly used techniques to measure membrane tethering. In most membrane tethering assays, liposomes are mixed with protein tethers that results in the clustering of vesicles. The resulting increase in the size of liposome clusters, as measured by dynamic light scattering (DLS), or an increase in the fluorescence intensity of liposomes as monitored by microscopy has been frequently used to assay for membrane tethering (Nakatogawa *et al.*, 2007; Ho and Stroupe, 2016; Segawa *et al.*, 2019). Therefore, we compared tethering at increasing concentrations of GST-FYVE using DLS and fluorescence microscopy-based liposome clustering with GLT. Extruded 400-nm liposomes containing PI3P were mixed with GST-FYVE at concentrations ranging from 0.5 to 4 µM. Fluorescence microscopy (Figure 3, A and B) and DLS (Figure 3C) revealed that larger clusters of liposomes could be observed with the addition of 0.5 µM of GST-FYVE. These clusters grew progressively larger in response to increasing GST-FYVE concentration and did not show a significant increase in size beyond 2 µM GST-FYVE (Figure 3, B and C). Similarly, GLT showed the recruitment of liposomes to GUVs with the addition of 0.5 µM of GST-FYVE and showed increasing tethering with increasing protein concentration (Figure 3, D and E). Although all of these techniques demonstrated tethering, nonsignificant differences in the amount of tethering were observed for all of the techniques over some concentration ranges. This indicates that while these techniques all demonstrate that membrane tethering occurred and was concentration-dependent, they have differential sensitivity across small changes in protein concentration. These results demonstrate that the sensitivity of GLT to detect tethering is comparable with DLS and fluorescence microscopy-based methods to measure membrane tethering.

### GLT can distinguish between membrane binding and tethering

**FIGURE 4:**
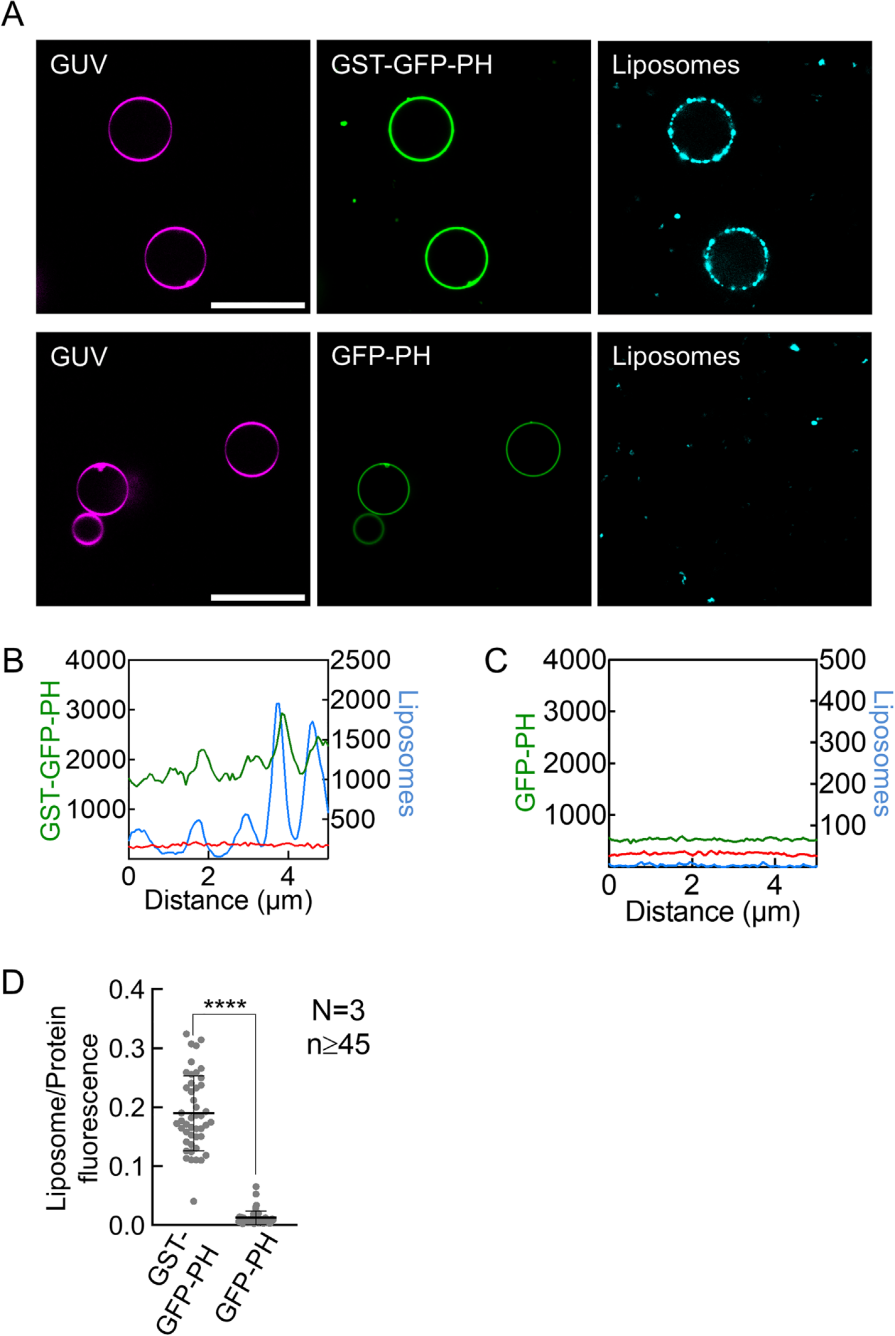
GLT of dimeric and monomeric PH domains with PI(4,5)P_2_ membranes. GUVs containing PI(4,5)P_2_ (magenta) were incubated with either GST-GFP-PH or GFP-PH and subsequently mixed with PI(4,5)P_2_-containing liposomes (cyan). (A) Representative fluorescence images of GLT where GUVs were mixed with liposomes and either GST-GFP-PH (top panel) or GFP-PH (bottom panel). (B) Fluorescence intensity profiles from a line drawn along the GUV in A (top panel) showing liposome and GST-GFP-PH fluorescence along the length of the GUV. (C) Fluorescence intensity profiles from a line drawn along the GUV in A (bottom panel) showing liposome and GFP-PH fluorescence along the length of the GUV. (D) Quantitation of A showing mean liposome fluorescence intensity from a segmented line drawn along the GUV surface normalized to mean protein fluorescence intensity. Data from three independent repeats (*N* = 3) were pooled and analyzed using nonparametric Mann–Whitney's test. ****p < 0.0001. *n* indicated total number of GUVs analyzed per condition. GUVs were made with DOPC (74.9 mol%), DOPS (20 mol%), DOPI(4,5)P_2_ (5 mol%), and RhPE (0.1 mol%). Liposomes were made with identical composition except RhPE that was replaced with DiD; scale bar, 20 µm.

One challenge in studying membrane tethering is differentiating between membrane binding and tethering. This often requires two assays: one to examine membrane binding and another to monitor membrane tethering. For example, the fluorescence microscopy and DLS assays used in Figure 3 only report on tethering and cannot detect protein–membrane binding that does not result in tethering. However, we reasoned that GLT could distinguish between membrane binding and tethering in a single experiment that would make it a powerful tool to combine with mutagenesis studies aimed to disrupt membrane binding or tethering. To test this, we purified GFP-EEA1 FYVE_1337-1411_ that lacks the dimerizing GST and is therefore a monomer that should not be able to tether membranes. However, without the dimerizing GST tag, this protein showed minimal membrane binding and was therefore not useful to distinguish between membrane binding and tethering (Supplemental Figure S2). This result is consistent with previous studies that showed the monomeric construct (amino acids, 1337–1411) of EEA1 FYVE has a very low affinity for PI3P (Dumas *et al.*, 2001). Instead, we used the monomeric pleckstrin homology (PH) domain of phospholipase C-delta 1 (PLC-delta) that has a high affinity for PI(4,5)P_2_ (Garcia *et al.*, 1995). We purified GFP-tagged PH domain with (GST-GFP-PH) and without (GFP-PH) the dimerizing GST tag and tested their abilities to tether membranes using GLT. Both constructs of the PH domain bound GUVs containing PI(4,5)P_2_, although GST-GFP-PH showed higher binding than GFP-PH that is consistent with an increased avidity for the membrane due to the dimerization of the two PH domains (Figure 4A). Liposomes were recruited to GUVs in the presence of the dimeric GST-GFP-PH, demonstrating that this construct can efficiently tether membranes. Fluorescence intensity traces along GUVs showed that the peaks in liposome fluorescence (cyan) were coincident with peaks in GST-GFP-PH fluorescence, further validating the use of GLT to monitor the presence of protein at membrane tethering events (Figure 4B). If the monomeric GFP-PH is capable of tethering liposomes to GUVs, albeit with less efficiency due to the reduced membrane binding of GFP-PH, then normalizing the integrated liposome fluorescence intensity to the GFP fluorescence intensity should give similar ratios between both GST-GFP-PH and GFP-PH. However, GST-GFP-PH had significantly higher liposome-to-protein fluorescence ratios as compared with GFP-PH that were all very close to zero, suggesting that essentially no liposomes were recruited to GUVs in the presence of GFP-PH (Figure 4, C and D). We therefore concluded that while GFP-PH retains membrane binding, it is incapable of tethering membranes, which would be expected for a monomeric PH domain. As such, these results demonstrate that GLT can be used to distinguish between membrane binding and tethering by proteins using a single assay.

### Calcium-induced fusion of tethered liposomes in GLT

**FIGURE 5:**
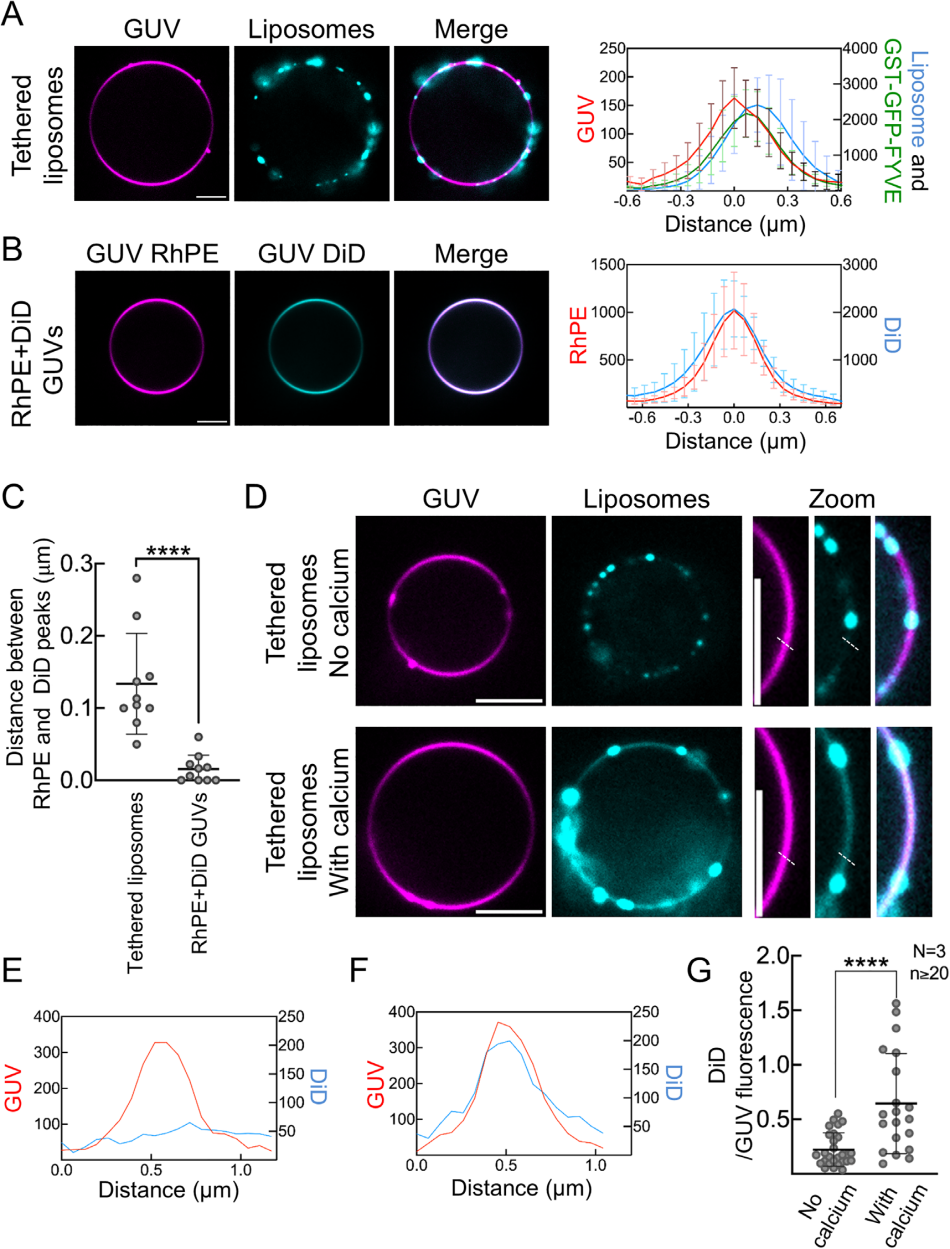
GLT can distinguish between membrane tethering and membrane fusion. Representative images showing (A) DiD-containing liposomes tethered to GST-GFP-FYVE-displaying RhPE GUVs, and (B) GUVs containing both RhPE and DiD. Fluorescence intensity profiles on the right represent the average intensity measured along a line drawn across the GUV from the lumen to the exterior. For panel A, the line was drawn across a region containing tethered liposomes. (C) Quantification of the distance between RhPE and DiD peaks derived from the fluorescence intensity profiles in panels A and B. Fluorescence intensity traces were fitted to Gaussian functions, and the distance between the RhPE and DiD peak maxima was calculated. (D) PI3P-containing liposomes were tethered to GUVs via GST-FYVE. Membrane fusion was then triggered by the addition of 15 mM calcium. Images were acquired in both the absence (top panel) and presence (bottom panel) of calcium. Fluorescence intensity profiles represent GUV and DiD signals from a line drawn across the GUV in a region without tethered liposomes (dotted white line) in the absence (E) and presence (F) of calcium. (G) Quantification of panel D showing DiD intensity normalized to GUV/RhPE intensity from areas devoid of tethered liposomes in the presence and absence of calcium. Data from three independent repeats (*N* = 3) were pooled and analyzed using nonparametric Mann–Whitney's test. ****p < 0.0001.

We next asked whether the GLT assay could be utilized to visualize membrane fusion. In principle, fusion of liposomes with GUVs would result in the appearance of DiD fluorescence within GUV membranes. As an initial test, we examined whether tethering of liposomes via the dimeric GST-FYVE protein alone was sufficient to induce fusion. Line-scan analysis from a line drawn perpendicular to a tethered liposome revealed spatially distinct fluorescence peaks corresponding to the GUV (RhPE) and liposome (DiD) signals (Figure 5A). In contrast, GUVs prepared with both RhPE and DiD incorporated together (RhPE+DiD GUVs) exhibited fully overlapping RhPE and DiD peaks (Figure 5B). Gaussian fitting of 10 such traces showed that the distance between RhPE and DiD peak maxima was greater for tethered liposomes than for RhPE+DiD GUVs (Figure 5C). Together, these results indicate that liposome tethering alone is insufficient to drive membrane fusion and mixing of DiD fluorescence into GUV membranes.

Next, we induced membrane fusion by calcium addition. Previous work has established that calcium promotes fusion of PS-containing membranes above a threshold of ∼0.35 to 0.39 Ca^2+^ per PS molecule (Duzgunes *et al.*, 1981; Roy and Sarkar, 2011; Grad *et al.*, 2024). We therefore added 15 mM calcium chloride, which is close to the predicted threshold for the 20% PS-containing membranes used in the GLT assay. Strikingly, DiD fluorescence appeared in regions between tethered liposomes following calcium addition (Figure 5D). Line-scan analysis from a line drawn perpendicular to the GUV in areas between tethered liposomes (white dotted lines in Figure 5D) revealed increased DiD fluorescence that overlapped with the GUV signal in the presence of calcium (Figure 5, E and F). Quantification of DiD intensity, normalized to the GUV signal in regions between tethered liposomes, further showed that calcium addition promotes mixing of DiD fluorescence with GUV membranes (Figure 5G). These results demonstrate that the GLT assay can also be utilized to study membrane fusion.

### GLT allows real-time visualization and kinetic analysis of membrane tethering

**FIGURE 6:**
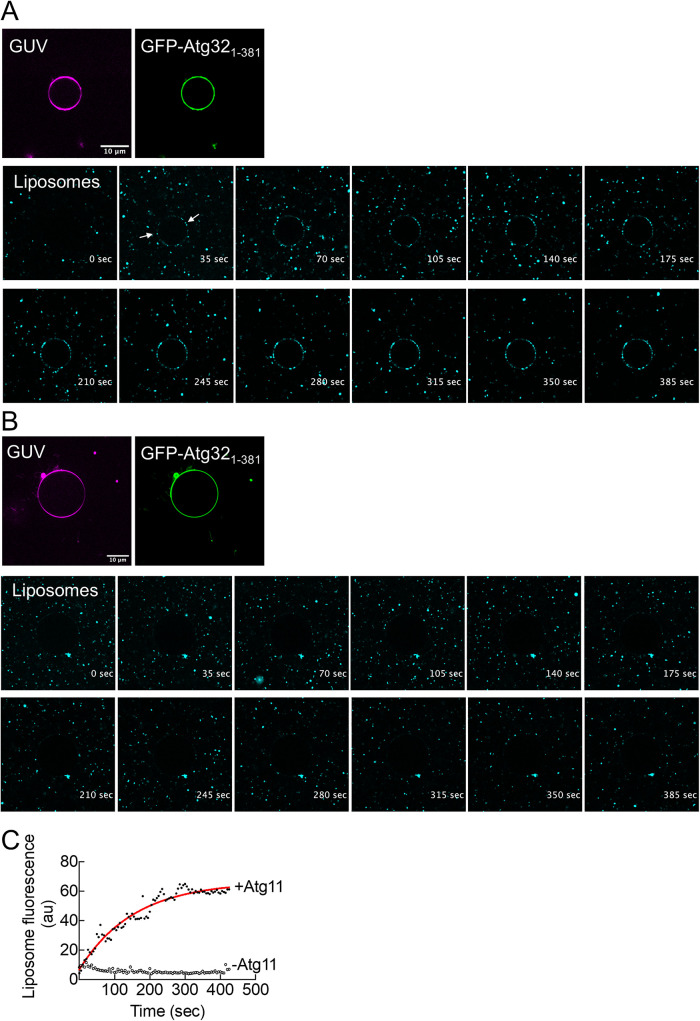
Real-time visualization and kinetic analysis of tethering using GLT. GUVs containing 5 mol% of DGS-Ni^+2^NTA incubated with GFP-Atg32_1-381_ and Atg11 or GFP-Atg32_1-381_ alone were allowed to settle in a LabTek well. A time-lapse series was recorded during the addition of YPL liposomes. (A) A montage showing the real-time addition and tethering of liposomes (cyan) to a GUV (magenta) bound to GFP-Atg32_1-381_(green) and Atg11. (B) Montage showing the real-time addition of liposomes (cyan) to a GUV (magenta) bound to GFP-Atg32_1-381_(green). (C) Representative trace showing the buildup of liposome fluorescence on a line drawn along the GFP-Atg32_1-381_ bound GUV surface in the presence (filled circles) and absence (empty circles) of Atg11. The red line shows the fit to a plateau followed by a one-phase exponential rise equation. GUVs were made with DOPC (74.9 mol%), DOPS (20 mol%), DGS-Ni^+2^NTA (5 mol%), and RhPE(0.1 mol%). Liposomes were made with YPL (99.9 mol%) and DiD (0.1 mol%).

During mitochondrial autophagy (mitophagy) in yeast, the outer mitochondrial membrane (OMM) protein Atg32 binds to Atg11 to tether autophagic vesicles to mitochondria. We previously demonstrated that Atg11 is recruited to GUVs only in the presence of Atg32 (1-381), and that Atg11 recruitment leads to tethering of autophagic liposomes (Andhare *et al.*, 2026). These previous results highlighted the ability of GLT to monitor heterotypic tethering events by protein complexes. Given the more physiological membrane tethering event by the Atg32–Atg11 complex, we decided to use this system to test whether GLT can be used to monitor membrane tethering in real-time. We attempted to follow Atg11-mediated tethering of liposomes made from yeast polar lipids (YPL) on GFP-Atg32_1-381_–bound GUVs. We found that after 10 min of addition, GUVs settled and were nonspecifically adhered to LabTek chamber wells. We added GFP-Atg32_1-381_–bound GUVs mixed with or without Atg11 on LabTek wells and allowed them to settle. Next, we started acquiring a time-lapse imaging sequence; added YPL liposomes and continued to monitor the fluorescence channel for the liposomes (Supplemental Movies S1 and S2). On GUVs displaying GFP-Atg32_1-381_ with Atg11, liposomes started tethering on the GUV surface at 20 s after the liposomes appeared in the background (Figure 6A; Supplemental Movie S1). Liposomes remained bound to GUVs, and liposome fluorescence on GUVs increased over time. In contrast, on GUVs displaying GFP-Atg32_1-381_ alone, liposomes did not bind GUVs and could be seen diffusing around GUVs throughout the length of the movie (Figure 6B; Supplemental Movie S2). Analysis of the build-up of liposome fluorescence on a line drawn along the circumference of the GUV confirmed that liposome fluorescence increased only in the presence of Atg11 (Figure 6C). The increase in liposome fluorescence in the presence of Atg11 fit to a plateau followed by a one-phase exponential rise equation and showed a *t*_half_ of 155 s. The average time for autophagosome completion in cells ranges from 110 s to several minutes, suggesting that tethering in the GLT assay occurs in a physiologically relevant timescale (Axe *et al.*, 2008; Broadbent *et al.*, 2023). These results further demonstrate that GLT can be used to monitor complex heterotypic membrane tethering events by protein complexes and establish that GLT can also be used to monitor the kinetics of these tethering events.

## DISCUSSION

We demonstrated the broad usefulness of GLT to study membrane tethering events in vitro using artificial membrane tethers made of dimeric membrane binding domains (GST-GFP-PH and GST-GFP-FYVE) that mediate homotypic tethering, and the mitophagy receptor Atg32 in complex with selective autophagy adaptor Atg11 that mediates heterotypic tethering. By using two different membranes with distinct fluorophores, we quantify the amount of membrane tethering by measuring liposome fluorescence along the circumference of individual GUVs, providing a more direct readout for membrane tethering than commonly used approaches that rely on liposome clustering.

There have been a few previous examples of laboratories using GUVs and liposomes to study membrane tethering (Drin *et al.*, 2008). However, these assays have primarily relied on representative images to demonstrate tethering and have not quantified the results over many GUVs. In contrast, we present the first systematic analysis of the GLT assay, examining lipid specificity, tethering reversibility, and fusion of tethered liposomes. We demonstrate that tethering in GLT, by proteins that bind lipid headgroups, occurs in a lipid-specific manner and that GLT offers comparable sensitivity with existing methods for studying membrane tethering, while providing the following significant advantages. First, GLT enables simultaneous monitoring of membrane tethering and the localization of individual protein components. This feature will be particularly valuable as more complex tethering sites are investigated, as it can highlight how efficiently different proteins are incorporated into membrane tethering sites. Second, GLT distinguishes membrane binding from tethering in a single assay, allowing mutations in tethering complex proteins to be tested directly for defects in membrane binding versus tethering. Third, GLT can be used to monitor vesicle fusion that may be occurring alongside tethering. Fourth, GLT enables real-time visualization of membrane tethering, making it possible to analyze subtle mutations or regulatory roles of proteins that only alter the kinetics of the process.

We previously used GLT to monitor the heterotypic tethering of autophagic-like liposomes to OMM-GUVs by the complex formed by Atg32 and Atg11 (Andhare *et al.*, 2026). In our previous work, we also demonstrated that full-length Atg11 recruited liposomes to Atg32-GUVs in a clustered manner, while just the N-terminal domain of Atg11 recruited liposomes in a uniform manner. To analyze this, we utilized the coefficient of variance of the liposome fluorescence along the length of the GUV. This highlighted that GLT can also be used to assess the organization and clustering of liposomes on the surface of GUVs, providing additional information about membrane tethering events.

Based on our demonstrated uses of GLT in this article and our previous studies (Hawkins *et al.*, 2022; Andhare *et al.*, 2026), we believe that GLT will be broadly useful for many laboratories that are studying membrane tethering. The requirements for performing this assay are simple; it requires access to a standard epifluorescence light microscope, the ability to generate GUVs and liposomes, and access to purified protein tethers. The most restricting aspect of the assay is generating GUVs. However, although many laboratories generate GUVs using electroformation, which requires specialized instrumentation (Angelova and Dimitrov, 1986; Okumura *et al.*, 2007; Bellon *et al.*, 2018), we generate GUVs for the GLT assay using the gel-assisted swelling method that does not require any specialized instrumentation (Weinberger *et al.*, 2013). Due to their size, GUVs are well-suited for imaging with a standard epifluorescence microscope, enabling GLT to be performed without access to a confocal microscope, adding to the accessibility of the technique. Additionally, all of our image analysis is performed using ImageJ that is freely available (Schindelin *et al.*, 2012).

The overall throughput of the GLT assay is limited by the number of GUVs that are produced and imaged throughout the experiment. Although the gel-assisted method used to generate GUVs yields a substantial number of vesicles, the overall yield depends on lipid composition, and only a few GUVs are typically visible within a single field of view at 100× magnification. Therefore, to ensure sufficient quantification of GUVs, it is essential to scan multiple fields of view to identify suitable numbers of GUVs for imaging and quantification. We typically aim to quantify at least 30 GUVs across three independent experiments that we have found is sufficient to capture the variability across an individual sample. Additionally, the use of multiwell imaging chambers enables multiple reactions to be performed in parallel, providing a straightforward path to scaling the assay for more high-throughput applications. Integration with automated image acquisition and analysis pipelines would further enable parallel imaging of multiple GLT reactions and unbiased quantification, thereby further enhancing the throughput of the assay.

To monitor tethering events, GLT quantifies the localization of liposomes on the surface of GUVs. Consequently, GLT is limited in its ability to investigate tethering when both membranes exhibit high curvature. GUVs are ideal for mimicking relatively flat membrane surfaces in cells such as the PM, ER, and mitochondria; while liposomes can be sonicated or extruded to mimic the curvature of vesicles, such as COP-II or clathrin-coated vesicles that are between 40 and 100 nm in diameter (Miller and Schekman, 2013; Kirchhausen *et al.*, 2014). This may make GLT optimal for reconstituting heterotypic tethering events where small vesicles are tethered to flat membranes before vesicle fusion, for example, exocyst-mediated heterotypic tethering of secretory vesicles to the PM (Lepore *et al.*, 2018).

In all of our GLT assays RhPE and DiD were used to label GUVs and liposomes, respectively. We showed that GST-FYVE tethered membranes do not undergo fusion on their own, but addition of fusogenic molecules, such as calcium can trigger fusion of DiD-containing liposomes with GUVs. This suggests that GLT could potentially be further adapted to directly visualize membrane fusion events by monitoring the exchange of fluorophores, making it an even more powerful technique to study membrane tethering and fusion.

## MATERIALS AND METHODS

Request a protocol through *Bio-protocol*

### Recombinant protein expression and purification

All constructs used for protein purification in this article are listed in Supplemental Table S1. The PH domain from GFP-C1-PLCdelta-PH was subcloned into pET 6xHis-GST-TEV LIC cloning vector (1G) (Addgene plasmid # 29655 that was a gift from Scott Gradia) to generate 12XHis-GST-GFP-PH. GFP-C1-PLCdelta-PH was a gift from Tobias Meyer (Addgene plasmid #21179) (Stauffer *et al.*, 1998). DNA coding for MTMR2 was synthesized and subcloned into the pET-28a(+) plasmid, generating an MTMR2 construct with a C-terminal TEV-6xHis tag. 6xHis-GST, 12XHis-GST-EEA1 FYVE, 12XHis-GST-GFP-EEA1 FYVE and 12XHis-GST-GFP-PH were recombinantly expressed in *Escherichia coli* Star cells (Invitrogen, C601003). Cultures were grown in LB broth (Thermo Fisher Scientific, BP1426) to an OD_600_ value of 0.6 to 0.8 at 37°C while shaking at 210 rpm. Cultures were cooled at 4°C for 20 min. Protein expression was induced by adding 1 mM isopropylthio-β-D-galactopyranoside (IPTG; IBI Scientific, IB02125), and the cultures were grown for an additional 18 h at 18°C. Cells were harvested, and cell pellets were stored at −80°C. MTMR2 was recombinantly expressed in BL21-CodongPlus (DE3)-RIPL cells (Agilent, 230280). Cultures were grown in LB broth (Thermo Fisher Scientific, BP1426) to an OD_600_ value of 0.6 to 0.8 at 37°C while shaking at 210 rpm. Cultures were cooled at RT for 20 min. Protein expression was induced by adding 0.2 mM isopropylthio-β-D-galactopyranoside (IPTG; IBI Scientific, IB02125), and the cultures were grown for an additional 18 h at 25°C. Cells were harvested and cell pellets were stored at −80°C.

For purification of 6xHis-GST, 12xHis-GST-EEA1 FYVE, and 12xHis-GST-GFP-EEA1 FYVE, cell pellets were thawed and resuspended in 50 mM Tris pH 8, 500 mM NaCl, 2.5 mM imidazole, 5 mM MgCl_2_, 1 mM phenylmethanesulfonyl fluoride (PMSF; VWR, 0754) and cOmplete Mini EDTA-free protease inhibitor tablets (Roche, 11836170001). Cells were lysed using three strokes of a microfluidizer at 18,000 psi. Triton X-100 (VWR, AAA16046-AE), was subsequently added to lysates at 1% vol/vol. Lysates were spun at 25,000 × *g* for 30 min. The supernatant was then applied to pre-equilibrated Talon resin (Clontech, 635504). The resin was washed with 50 mM Tris, pH 8, 500 mM NaCl, 10 mM imidazole. Bound proteins were eluted with 50 mM Tris pH8, 500 mM NaCl, 250 mM imidazole. For purification of 6xHis-GST, 12xHis-GST-EEA1 FYVE, 12xHis-GST-GFP-EEA1, peak fractions containing protein were pooled and dialyzed against 20 mM Tris pH 8, 300 mM NaCl, and 0.2 mM TCEP. Proteins were aliquoted, flash-frozen in liquid nitrogen, and stored at −80°C until use.

For purification of 12XHis-GST-GFP-PH constructs, cell pellets were resuspended and thawed in 20 mM Tris, pH 8.0, 500 mM NaCl, 2.5 mM imidazole, 1 mM DTT, and a cOmplete Mini protease inhibitor tablet (Roche, 11836170001). Cells were lysed using three strokes of a microfluidizer at 18,000 psi. Triton X-100 (VWR, AAA16046-AE) was subsequently added to lysates at 1% vol/vol. Lysates were spun at 25,000 × *g* for 30 min. Supernatant was added to pre-equilibrated GST resin and incubated on a rocker for 1.5 h at 4°C. Resin was washed with 20 mM Tris, pH 8.0, 500 mM NaCl. The GST resin was divided into two parts; one part was used for elution of GST-GFP-PH using 20 mM Tris, pH 8.0, 300 mM NaCl, and 40 mM reduced glutathione. Eluted protein was dialyzed against 20 mM Tris, pH 8.0, 300 mM NaCl, and 0.2 mM TCEP. The other half of the GST resin was incubated with TEV overnight at 4°C. Flow-through from the GST resin containing GFP-PH was subsequently incubated with Talon resin to remove TEV. Flow-through from the Talon resin was subjected to size exclusion chromatography using a Superdex 75 column. Peak fractions containing proteins were aliquoted, flash-frozen in liquid nitrogen, and stored at −80°C until use.

GFP-Atg32_1-381_ and Atg11 were purified as described previously (Andhare *et al.*, 2026).

For purification of MTMR2, cell pellets were resuspended and thawed in 50 mM Tris, pH 8.0, 500 mM NaCl, 1% Triton X-100 (VWR, AAA16046-AE), 5 mM MgCl_2_, 1 mM phenylmethanesulfonyl fluoride (PMSF; VWR, 0754), and cOmplete Mini EDTA-free protease inhibitor tablets (Roche, 11836170001). Cells were lysed using three strokes of a microfluidizer at 18,000 psi. Lysates were spun at 75,000 × *g* for 45 min at 4°C. The supernatant was then applied to pre-equilibrated Talon resin (Clontech, 635504). The resin was washed with lysis buffer, wash buffer 1 (50 mM Tris, pH 8, 500 mM NaCl, and 2.5 mM imidazole), and wash buffer 2 (50 mM Tris, pH 8, 500 mM NaCl, and 2.5 mM imidazole). Bound proteins were eluted with 50 mM Tris, pH 8, 150 mM NaCl, and 250 mM imidazole. Elution fractions containing protein were pooled and further purified by size exclusion chromatography using a Superdex 200 column equilibrated in 20 mM Tris, pH 8.0, 150 mM NaCl, and 0.2 mM TCEP. Fractions containing protein were aliquoted, flash-frozen in liquid nitrogen and stored at −80°C until use.

### Liposome preparation

All lipids were purchased from Avanti Polar Lipids. Chloroform stocks of lipid mixtures at a desired ratio containing 0.1 mol% of the fluorescent lipophilic dye DiD were aliquoted in a clean glass tube and dried under a gentle nitrogen stream until all the chloroform had evaporated. Residual chloroform was removed under vacuum for 18 h. Dried lipids were resuspended in 20 mM Tris, pH 8, 150 mM NaCl to a final concentration of 1 mM. Liposomes were prepared by extrusion through 400-nm Nuclepore track-etched membrane filters (Sigma-Aldrich) using an Avanti Mini-Extruder (Avanti Polar Lipids).

### Giant unilamellar vesicle preparation

GUVs were made using the gel-assisted formation method (Weinberger *et al.*, 2013). Briefly, chloroform stocks containing lipids mixed at desired ratios containing the fluorescent lipid RhPE were aliquoted in a glass tube. Chloroform was dried under a stream of nitrogen, followed by drying under vacuum (1 h). The dried lipids were resuspended in fresh chloroform to make a 1-mM lipid mixture. Polyvinyl alcohol (PVA, Mw = 14,500; Sigma, 814894) was dissolved in boiling water to make a 5% stock and degassed in a vacuum chamber. PVA solution (10 µl) was spread into a thin film on a glass coverslip kept on a heating block set to 55°C. Chloroform-dissolved lipid mix (10 µl) was then spread on the dried PVA film. The dried PVA-lipid films were peeled from the glass coverslips and transferred to a 1.5-mL microcentrifuge tube. Films were hydrated in 300 µl assay buffer (20 mM Tris, pH 7.4, 150 mM NaCl) for 20 min, and GUVs were released by gentle tapping. The PVA films were removed using a pipette.

### GUV liposome tethering

For GUV liposome tethering (GLT) experiments, GUVs were first mixed with a tethering protein or control protein in a 1.5 ml Eppendorf tube for 10 min at room temperature. GUV mixtures were tapped to resuspend settled GUVs and subsequently incubated with 10 µl of 400-nm extruded liposomes for 5 min at room temperature. Liposomes were used within 3 d of extrusion. The GLT mixtures (300µL total volume) were transferred to a BSA-passivated LabTek chamber for imaging. GUVs were allowed to settle for 5 min before imaging. LabTek chambers were treated with 3 M NaOH for 10 min, followed by extensive washes with milliQ water. NaOH-treated chambers were then passivated by incubation with BSA (3 mg/ml). BSA was removed by three washes with the assay buffer.

To induce membrane fusion, GLT mixtures were further incubated with 15 mM calcium chloride for 5 min and imaged similarly.

### DLS assay and microscopy-based liposomes clustering assay

To monitor liposome tethering using DLS, GST-FYVE was spun at 100,000 × *g* for 20 min to remove large aggregates. A total of 50 µL of PI3P-containing 400 nm extruded liposomes were mixed with increasing concentrations of GST-FYVE in a total volume of 80 µL. The liposome and GST-FYVE mixture was placed on a rocker and incubated for 30 min at room temperature. Liposome size was measured using DynaPro NanoStar.

To measure liposome clustering, PI3P-containing liposomes (with 0.1 mol% of DiD) mixed with GST-FYVE prepared as described above were added to a BSA-passivated LabTek chamber. Liposomes were allowed to settle for 5 min and 10 fields were randomly imaged for each GST-FYVE concentration.

### Fluorescence microscopy

GLT and liposome clustering were imaged using a Nikon TiE microscope fitted with a Yokogawa CSU-W1 spinning disk system using a 100 X, 1.45 NA oil immersion objective. Images were acquired with a Photometrics Prime BSI sCMOS camera using the NIS Elements software.

For real-time analysis of tethering, time-lapse images in the liposome fluorescence channel (DiD) were acquired at every 5-s interval for 7 min.

### Image analysis

Fluorescence microscopy images were analyzed using Fiji (Schindelin *et al.*, 2012) and statistical analysis was performed using GraphPad Prism (version 5.0a).

All experiments were performed in at least triplicate. Data from three independent repeats (*N* = 3) were pooled and plotted as scatterplots. Error bars represent SD.

A percentage liposome fluorescence per length of GUV was calculated by normalizing the liposome fluorescence, collected from a two-pixel-wide segmented line drawn along a GUV, to the length of the GUV. For calculating the liposome/protein fluorescence ratio, fluorescence intensities in the liposome and protein channels from a two-pixel-wide segmented line drawn along a GUV were collected. Mean liposome fluorescence was divided by mean protein fluorescence to yield liposome/protein fluorescence ratios.

To analyze tethering using the microscopy-based liposome clustering assay, images were first background corrected using the mode intensity value. Images were auto-thresholded. Integrated density values of all particles with an area more than 0.1257 µm^2^ were plotted.

## Supporting information





## Data Availability

All of the data from this article are available on the Dartmouth Dataverse at doi.org/10.21989/D9/HNN67K.

## Abbreviations used:

COPII: Coat protein complex II
CORVET: Class C core vacuole/endosome tethering
DLS: Dynamic light scattering
DOPC: 1,2-dioleoyl-sn-glycero-3-phosphocholine
DOPS: 1,2-dioleoyl-sn-glycero-3-phospho-L-serine
EEA1: Early Endosome Antigen 1
ER: Endoplasmic reticulum
E-Syt: Extended synaptotagmin
FYVE: Fab1p, YOTB, Vac1p, and EEA1
GFP: Green fluorescent protein
GLT: Giant unilamellar vesicle and liposome tethering assay
GST: Glutathione S-transferase
GUV: Giant unilamellar vesicles
HOPS: Homotypic fusion and vacuole protein sorting
MTMR2: Myotubularin-related protein 2
OMM: Outer mitochondrial membrane
PH: Pleckstrin homology
PI3P: Phosphatidylinositol-3-phosphate
PM: Plasma membrane
RAB: Ras-related in brain
RhPE: Rhodamine phosphatidylethanolamine
SNARE: Soluble NSF Attachment Protein Receptor.

## References

[B1] Andhare D, Katzenell S, Najera SI, Mauras SC, Bauer KM, Ragusa MJ (2026). Reconstitution of autophagic-like membrane tethering reveals that Atg11 can bind and cluster vesicles on cargo mimetics. Autophagy 22, 484–503. 40899612 10.1080/15548627.2025.2551678 PMC12453139

[B2] Angelova MI, Dimitrov DS (1986). Liposome electroformation. Faraday Discuss 81, 303–311.

[B3] Axe EL, Walker SA, Manifava M, Chandra P, Roderick HL, Habermann A, Griffiths G, Ktistakis NT (2008). Autophagosome formation from membrane compartments enriched in phosphatidylinositol-3-phosphate and dynamically connected to the endoplasmic reticulum. J Cell Biol 182, 685–701. 18725538 10.1083/jcb.200803137 PMC2518708

[B4] Begley MJ, Taylor GS, Kim SA, Veine DM, Dixon JE, Stuckey JA (2003). Crystal structure of a phosphoinositide phosphatase, MTMR2: insights into myotubular myopathy and Charcot–Marie–Tooth syndrome. Mol Cell 12, 1391–1402. 14690594 10.1016/s1097-2765(03)00486-6

[B5] Bellon JA, Pino MJ, Wilke N (2018). Low-cost equipment for electroformation of Giant Unilamellar Vesicles. Hardwarex 4, e00037.

[B6] Bian X, Saheki Y, De Camilli P (2018). Ca(2+) releases E-Syt1 autoinhibition to couple ER-plasma membrane tethering with lipid transport. EMBO J 37, 219–234. 29222176 10.15252/embj.201797359 PMC5770786

[B7] Broadbent DG, Barnaba C, Perez GI, Schmidt JC (2023). Quantitative analysis of autophagy reveals the role of ATG9 and ATG2 in autophagosome formation. J Cell Biol 222, e202210078. 37115157 10.1083/jcb.202210078 PMC10148237

[B8] Drin G, Morello V, Casella JF, Gounon P, Antonny B (2008). Asymmetric tethering of flat and curved lipid membranes by a golgin. Science 320, 670–673. 18451304 10.1126/science.1155821

[B9] Dumas JJ, Merithew E, Sudharshan E, Rajamani D, Hayes S, Lawe D, Corvera S, Lambright DG (2001). Multivalent endosome targeting by homodimeric EEA1. Mol Cell 8, 947–958. 11741531 10.1016/s1097-2765(01)00385-9

[B10] Duzgunes N, Nir S, Wilschut J, Bentz J, Newton C, Portis A, Papahadjopoulos D (1981). Calcium- and magnesium-induced fusion of mixed phosphatidylserine/phosphatidylcholine vesicles: effect of ion binding. J Membr Biol 59, 115–125. 7241577 10.1007/BF01875709

[B11] Eisenberg-Bord M, Shai N, Schuldiner M, Bohnert M (2016). A tether is a tether is a tether: tethering at membrane contact sites. Dev Cell 39, 395–409. 27875684 10.1016/j.devcel.2016.10.022

[B12] Garcia P, Gupta R, Shah S, Morris AJ, Rudge SA, Scarlata S, Petrova V, McLaughlin S, Rebecchi MJ (1995). The pleckstrin homology domain of phospholipase C-delta 1 binds with high affinity to phosphatidylinositol 4,5-bisphosphate in bilayer membranes. Biochemistry 34, 16228–16234. 8519781 10.1021/bi00049a039

[B13] Grad P, Edwards K, Gedda L, Hernández VA (2024). A closer look at calcium-induced interactions between phosphatidylserine-(PS) doped liposomes and the structural effects caused by inclusion of gangliosides or polyethylene glycol-(PEG) modified lipids. Biochim Biophys Acta Biomembr 1866, 184253. 37979667 10.1016/j.bbamem.2023.184253

[B14] Hawkins WD, Leary KA, Andhare D, Popelka H, Klionsky DJ, Ragusa MJ (2022). Dimerization-dependent membrane tethering by Atg23 is essential for yeast autophagy. Cell Rep 39, 110702. 35443167 10.1016/j.celrep.2022.110702 PMC9097366

[B15] Ho R, Stroupe C (2016). The HOPS/class C Vps complex tethers high-curvature membranes via a direct protein–membrane interaction. Traffic 17, 1078–1090. 27307091 10.1111/tra.12421

[B16] Huang X, Jiang C, Yu L, Yang A (2020). Current and emerging approaches for studying inter-organelle membrane contact sites. Front Cell Dev Biol 8, 195. 32292782 10.3389/fcell.2020.00195 PMC7118198

[B17] Jahn R, Scheller RH (2006). SNAREs–engines for membrane fusion. Nat Rev Mol Cell Biol 7, 631–643. 16912714 10.1038/nrm2002

[B18] Jing J, Liu G, Huang Y, Zhou Y (2020). A molecular toolbox for interrogation of membrane contact sites. J Physiol 598, 1725–1739. 31119749 10.1113/JP277761 PMC7098838

[B19] Kirchhausen T, Owen D, Harrison SC (2014). Molecular structure, function, and dynamics of clathrin-mediated membrane traffic. Cold Spring Harb Perspect Biol 6, a016725. 24789820 10.1101/cshperspect.a016725 PMC3996469

[B20] Lepore DM, Martinez-Nunez L, Munson M (2018). Exposing the elusive Exocyst structure. Trends Biochem Sci 43, 714–725. 30055895 10.1016/j.tibs.2018.06.012 PMC6108956

[B21] Lurick A, Kummel D, Ungermann C (2018). Multisubunit tethers in membrane fusion. Curr Biol 28, R417–R420. 29689226 10.1016/j.cub.2017.12.012

[B22] Miller EA, Schekman R (2013). COPII—a flexible vesicle formation system. Curr Opin Cell Biol 25, 420–427. 23702145 10.1016/j.ceb.2013.04.005 PMC3736695

[B23] Nakatogawa H, Ichimura Y, Ohsumi Y (2007). Atg8, a ubiquitin-like protein required for autophagosome formation, mediates membrane tethering and hemifusion. Cell 130, 165–178. 17632063 10.1016/j.cell.2007.05.021

[B24] Okumura Y, Zhang H, Sugiyama T, Iwata Y (2007). Electroformation of giant vesicles on a non-electroconductive substrate. J Am Chem Soc 129, 1490–1491. 17283981 10.1021/ja068127x

[B25] Phillips MJ, Voeltz GK (2016). Structure and function of ER membrane contact sites with other organelles. Nat Rev Mol Cell Biol 17, 69–82. 26627931 10.1038/nrm.2015.8 PMC5117888

[B26] Roy SM, Sarkar M (2011). Membrane fusion induced by small molecules and ions. J Lipids 2011, 528784. 21660306 10.1155/2011/528784 PMC3108104

[B27] Saheki Y, De Camilli P (2017). The Extended-Synaptotagmins. Biochim Biophys Acta Mol Cell Res 1864, 1490–1493. 28363589 10.1016/j.bbamcr.2017.03.013 PMC5642939

[B28] Schaletzky J, Dove SK, Short B, Lorenzo O, Clague MJ, Barr FA (2003). Phosphatidylinositol-5-phosphate activation and conserved substrate specificity of the myotubularin phosphatidylinositol-3-phosphatases. Curr Biol 13, 504–509. 12646134 10.1016/s0960-9822(03)00132-5

[B29] Schindelin J, Arganda-Carreras I, Frise E, Kaynig V, Longair M, Pietzsch T, Preibisch S, Rueden C, Saalfeld S, Schmid B, *et al.* (2012). Fiji: An open-source platform for biological-image analysis. Nat Methods 9, 676–682. 22743772 10.1038/nmeth.2019 PMC3855844

[B30] Scorrano L, De Matteis MA, Emr S, Giordano F, Hajnoczky G, Kornmann B, Lackner LL, Levine TP, Pellegrini L, Reinisch K, *et al.* (2019). Coming together to define membrane contact sites. Nat Commun 10, 1287. 30894536 10.1038/s41467-019-09253-3 PMC6427007

[B31] Segawa K, Tamura N, Mima J (2019). Homotypic and heterotypic trans-assembly of human Rab-family small GTPases in reconstituted membrane tethering. J Biol Chem 294, 7722–7739. 30910814 10.1074/jbc.RA119.007947 PMC6514636

[B32] Shvarev D, Schoppe J, Konig C, Perz A, Fullbrunn N, Kiontke S, Langemeyer L, Januliene D, Schnelle K, Kummel D, *et al.* (2022). Structure of the HOPS tethering complex, a lysosomal membrane fusion machinery. Elife 11. 10.7554/eLife.80901 PMC9592082 36098503

[B33] Song H, Wickner W (2019). Tethering guides fusion-competent trans-SNARE assembly. Proc Natl Acad Sci USA 116, 13952–13957. 31235584 10.1073/pnas.1907640116 PMC6628791

[B34] Spang A (2016). Membrane tethering complexes in the endosomal system. Front Cell Dev Biol 4, 35. 27243003 10.3389/fcell.2016.00035 PMC4860415

[B35] Stauffer TP, Ahn S, Meyer T (1998). Receptor-induced transient reduction in plasma membrane PtdIns(4,5)P2 concentration monitored in living cells. Curr Biol 8, 343–346. 9512420 10.1016/s0960-9822(98)70135-6

[B36] Stroupe C, Hickey CM, Mima J, Burfeind AS, Wickner W (2009). Minimal membrane docking requirements revealed by reconstitution of Rab GTPase-dependent membrane fusion from purified components. Proc Natl Acad Sci USA 106, 17626–17633. 19826089 10.1073/pnas.0903801106 PMC2764952

[B37] Szentgyörgyi V, Spang A (2023). Membrane tethers at a glance. J Cell Sci 136, jcs260471. 36876970 10.1242/jcs.260471

[B38] Ueda S, Tamura N, Mima J (2020). Membrane tethering potency of Rab-family small GTPases is defined by the C-terminal hypervariable regions. Front Cell Dev Biol 8, 577342. 33102484 10.3389/fcell.2020.577342 PMC7554592

[B39] Ungermann C, Kümmel D (2019). Structure of membrane tethers and their role in fusion. Traffic 20, 479–490. 31062920 10.1111/tra.12655

[B40] Weinberger A, Tsai FC, Koenderink GH, Schmidt TF, Itri R, Meier W, Schmatko T, Schroder A, Marques C (2013). Gel-assisted formation of giant unilamellar vesicles. Biophys J 105, 154–164. 23823234 10.1016/j.bpj.2013.05.024 PMC3699747

