# Supplementary material for: A GUV-based assay to reconstitute membrane tethering in vitro
Source: Mol Biol Cell. 2026 Jun 10;37(7):mr7. doi: 10.1091/mbc.E25-11-0531 (PMC13329846; doi:10.1091/mbc.E25-11-0531)
Supplement: Supplementary file 1 [file mbc-37-mr7-s001.pdf]

Supplemental Materials

Molecular Biology of the Cell

Andhare *et al*.

Supplemental Materials for

A GUV-based assay to reconstitute membrane tethering in vitro.

Devika Andhare and Michael J Ragusa

Department of Chemistry, Dartmouth College, Hanover, NH 03755, USA

Table S1. List of constructs for protein purification used in this study.

**Uniprot ID**	**Protein expressed**	**Construct**	**Vector**	**Expression System**
Q15075	GST-EEA1-FYVE	12xHis-GST-TEV-EEA1 FYVE (1347-1411)	1G	*E coli*
Q15075	GST-GFP-EEA1-FYVE	12xHis-GST-TEV-GFP-EEA1 FYVE (1347-1411)	1G	*E coli*
P51178	GSt-GFP-PLCδPH	12xHis-GST-TEV-GFP-PLCδPH	1G	*E coli*
P40458	Atg32	StrepII-GFP-Atg32 (1 to 381)-10xHis	pET52	*E coli*
Q12527	Atg11	2xStrepII-HRV3C-Atg11	pCMV1	*Freestyle*
Q13614-1	MTMR2	MTMR2-TEV-6xHis	pET28	*E coli*

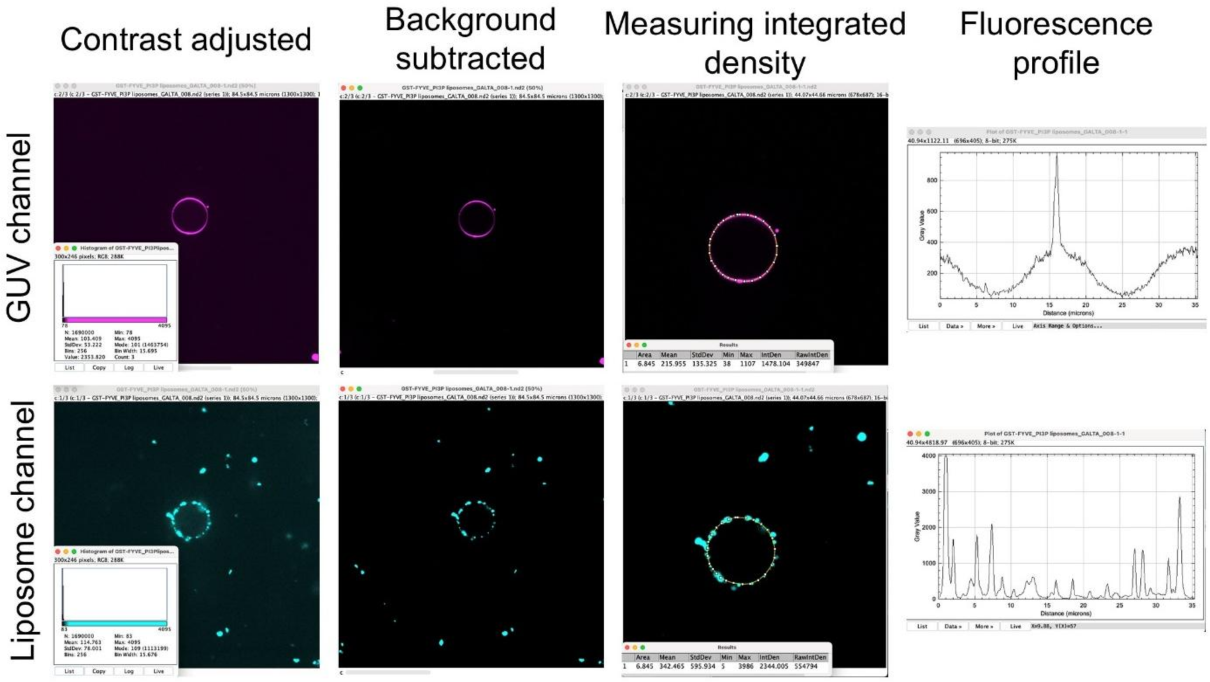

Figure S1. The general pipeline for GLT data analysis. Images are opened in Image J, contrast adjusted, and background subtracted. Individual GUVs are highlighted using the segmented line tool and the integrated density is measured. The ratio of liposome integrated density to GUV circumference or to tethering protein integrated density (when available) is then calculated for individual GUVs across three independent repeats.

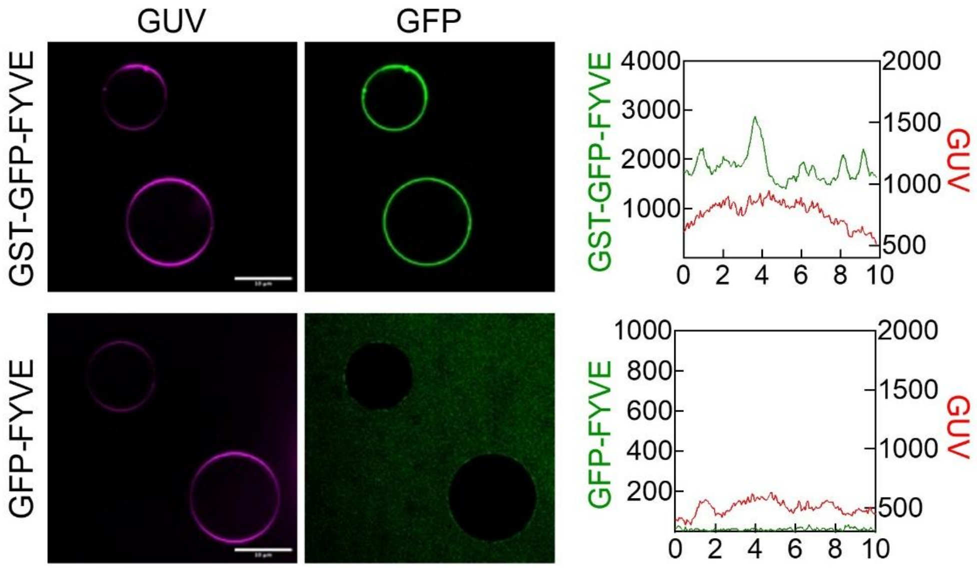

Figure S2. Monomeric GFP-FYVE is unable to bind membranes. Representative fluorescence images showing GST-GFP-FYVE (top) and GFP-FYVE (bottom) added to PI3P containing GUVS (74.9 mol% DOPC, 20 mol% DOPS, 5 mol% PI3P and 0.1 mol% RhPE). Fluorescence intensity traces on the right of the images show GST-GFP-FYVE and GFP-FYVE intensities along a GUV. Scale bar=10 μm.
